# Comparison of organ dysfunction patterns in pediatric hemophagocytic lymphohistiocytosis and sepsis: incidence, adverse outcomes, and cluster characteristics

**DOI:** 10.3389/fped.2025.1708908

**Published:** 2025-11-13

**Authors:** Jinpeng Gan, Xun Li, Haipeng Yan, Xiao Li, Xiangyu Wang, Longlong Xie, Ting Luo, Yufan Yang, Haixia Yang, Haiyan Luo, Xinping Zhang, Jiaotian Huang, Zhenghui Xiao, Xiulan Lu

**Affiliations:** 1 The School of Pediatrics, Hengyang Medical School, University of South China, Changsha, China; 2 Pediatrics Research Institute of Hunan Province, Affiliated Children’s Hospital of Xiangya School of Medicine, Central South University (Hunan Children’s Hospital), Changsha, China; 3 Pediatric Intensive Care Unit, Affiliated Children’s Hospital of Xiangya School of Medicine, Central South University (Hunan Children’s Hospital), Changsha, China; 4 Hunan Provincial Key Laboratory of Emergency Medicine for Children, Affiliated Children’s Hospital of Xiangya School of Medicine, Central South University (Hunan Children’s Hospital), Changsha, China; 5 International Inpatient Ward, Affiliated Children’s Hospital of Xiangya School of Medicine, Central South University (Hunan Children’s Hospital), Changsha, China; 6 Department of Radiology, Affiliated Children’s Hospital of Xiangya School of Medicine, Central South University (Hunan Children’s Hospital), Changsha, China; 7 Department of Pediatric Hematology, Affiliated Children’s Hospital of Xiangya School of Medicine, Central South University (Hunan Children’s Hospital), Changsha, China

**Keywords:** organ dysfunction, children, hemophagocytic lymphohistiocytosis, multipleorgan dysfunction syndrome, sepsis, septic shock, pediatric intensive care

## Abstract

**Background:**

Hemophagocytic lymphohistiocytosis (HLH) and severe sepsis share similarities in their clinical manifestations, and both have high risks of developing multiple organ dysfunction syndrome (MODS). The aim of this study was to investigate the similarities and differences in organ dysfunction patterns among pediatric patients with HLH and severe sepsis.

**Methods:**

Pediatric patients diagnosed with either HLH or severe sepsis from a tertiary children's hospital over a 5-year period were included. Eleven complications representing organ dysfunction in major systems (hepatic, cardiovascular, respiratory, renal, hematologic, neurologic, and gastrointestinal) were examined. The primary outcome was adverse outcome, defined as in-hospital death or discharge following withdrawal of advanced life-sustaining treatment. The incidence and adverse outcome rates of organ dysfunction among pediatric patients with HLH and severe sepsis were compared, along with the cumulated number of complications and the correlation networks of complications, as well as laboratory characteristics.

**Results:**

This study included 231 pediatric patients with HLH and 259 with severe sepsis. Adverse outcomes occurred in 15.2% of HLH patients and 18.9% of severe sepsis patients. In HLH, the most prevalent complications were hepatic injury (46.8%) and coagulopathy (43.3%), while adverse outcome rates were highest among patients who developed ARDS (81.8%) and heart failure (77.8%). In severe sepsis, the leading complications were shock (69.9%), respiratory failure (52.1%), and coagulopathy (51.0%); adverse outcome rates were highest among patients with heart failure (67.5%), hepatic failure (61.9%), and ARDS (50%). For most complications investigated, HLH showed lower incidences compared to severe sepsis, but with similar or higher adverse outcome rates. Under the same number of complications, HLH had a higher adverse outcome rates than severe sepsis. However, patients with severe sepsis tended to develop more complications (median 3 vs. 2, *P* < 0.0001), resulting in similar overall adverse outcome rates for these two conditions.

**Conclusions:**

The incidence, adverse outcome rates, and clustering patterns of organ dysfunction differed between HLH and severe sepsis. Strategies to improve prognosis should vary for each condition. In HLH, preventing the development of severe organ dysfunction is crucial, whereas in severe sepsis, the emphasis should be on preventing the clustering of multiple complications.

## Background

Hemophagocytic lymphohistiocytosis (HLH) and sepsis are both severe and life-threatening conditions encountered in the Pediatric Intensive Care Unit (PICU). HLH is a hyperinflammatory syndrome characterized by excessive activation of the immune system (1). The uncontrolled proliferation of activated macrophages and lymphocytes resulting in cytokine storm can lead to multiple organ dysfunction syndrome (MODS). While the definition of sepsis has evolved over time, including the 2005 International Pediatric Sepsis Consensus Conference (2), the 2012 Surviving Sepsis Campaign guidelines (3), and the recent Phoenix criteria (4), it fundamentally refers to life-threatening organ dysfunction caused by a dysregulated host response to infection, characterized by widespread inflammation, endothelial dysfunction, and coagulation abnormalities. Severe sepsis has been commonly used to describe sepsis accompanied by cardiovascular organ dysfunction, acute respiratory distress syndrome, or dysfunction of two or more other organ systems (2). HLH and sepsis share similarities in their clinical manifestations, such as unbalanced immune responses, making early differential diagnosis challenging (5–8). However, the underlying mechanisms of these conditions differ significantly, which in turn influences their respective treatment approaches. The treatment for HLH typically involves immunosuppressive therapy to counteract the overactive immune response (9, 10). On the other hand, the treatment for sepsis and its severe forms focuses on addressing the underlying infection, supporting organ function, and managing the dysregulated host response (3). Although some supportive treatment procedures may be beneficial for both patient groups, distinct management strategies for HLH and sepsis are critical for patient outcomes.

Efforts have been made to find methods to distinguish HLH from sepsis. Machowicz et al. summarized the clinical and laboratory characteristics of HLH and sepsis, revealing that parameters such as hyperferritinemia, splenomegaly, pronounced cytopenias, hypofibrinogenemia, low C-reactive protein (CRP), and a characteristic cytokine profile are helpful in discriminating HLH from sepsis (11). Lin et al. investigated the concentration of 135 inflammatory plasma proteins in patients with HLH, sepsis, and Systemic Inflammatory Response Syndrome (SIRS) (12). They found that 15 proteins were significantly different in HLH compared to SIRS/sepsis. Subsequently, they developed a plasma protein classifier, which included CXCL9 and interleukin-6, to differentiate HLH from SIRS/sepsis. Li et al. compared the plasma proteomic profiles between HLH and sepsis and identified 28 differentially expressed proteins (13). These proteins were mainly involved in pathways related to neutrophil extracellular trap formation, platelet activation, and fluid shear stress, as well as atherosclerosis. Chaturvedi et al. examined T-cell profiles from children with either HLH or sepsis and found that activated T cells in HLH is characterized by CD38^high^/HLA-DR1^+^ effector cells (14). While the differential diagnosis of HLH and sepsis has become clearer, there remains a lack of information regarding the differences in organ dysfunction features between the two conditions. Both HLH and sepsis have high risks of developing MODS (15, 16), yet the profiles of organ dysfunction may differ due to their distinct mechanisms. Identifying the similarities and differences in their organ dysfunction patterns can aid in clinical management, including monitoring, treatment, and prevention of deterioration.

This study aims to investigate the similarities and differences in the patterns of organ dysfunction among pediatric patients with HLH and severe sepsis by comparing their incidence and rates of adverse outcome, analyzing complication clustering and networks, and examining laboratory characteristics. Severe sepsis is marked by more pronounced clinical symptoms and a higher risk of complications compared to its less severe forms (3). Studying severe sepsis allows for a more fitting comparison with HLH in terms of severity, clinical management challenges, and outcomes. Therefore, this study focuses on severe sepsis rather than encompassing all forms of sepsis. We also explored the complication profiles by disease subgroups, categorized according to the types and potential triggers of HLH and subdivided severe sepsis into with and without septic shock.

## Materials and methods

### Study population and group allocation

Patients from Hunan Children's Hospital who were diagnosed with either HLH or severe sepsis between January 2018 and July 2023 were included in this study. Chart review and data collection were conducted between September 2023 and October 2023. HLH was diagnosed using the HLH-2004 criteria (10). Severe sepsis and septic shock were diagnosed according to the 2005 International Pediatric Sepsis Consensus Conference definitions (2). Patients were allocated to either the HLH group or the severe sepsis group according to their main diagnosis. If a patient had been diagnosed with both HLH and sepsis, he or she was allocated to the HLH group. The exclusion criteria were: patient age over 18 and an undetermined diagnosis of HLH or severe sepsis. The HLH group was further subdivided according to the types and potential triggers of HLH, including primary HLH, EBV-associated, other infection-associated, malignancy-associated, autoimmune disease-associated HLH, and other/undetermined types of HLH. The severe sepsis group was subdivided into septic shock and severe sepsis without septic shock. The subgroup allocation was determined based on the diagnosis from the medical record and was reviewed by two senior physicians. The observation window for this study was defined as the period from hospital admission to hospital discharge. All complications and outcomes were recorded during this hospitalization period. The study protocol was reviewed and approved by the Medical Ethics Committee of the Hunan Children's Hospital (HCHLL-2023-97).

We acknowledge that the Phoenix criteria for pediatric sepsis have been recently adopted (4). However, our study period (2018–2023) predates the widespread implementation of these criteria in clinical practice. The use of the 2005 International Pediatric Sepsis Consensus Conference definitions (2) reflects the diagnostic standards applied during the study period and may affect the generalizability of our findings to settings using newer definitions.

### Standard treatment protocols

In our institution, HLH treatment followed the HLH-94 protocol (9) with immunosuppressive and supportive therapy. Sepsis management adhered to the 2012 Surviving Sepsis Campaign guidelines (3), emphasizing early antimicrobial therapy, source control, fluid resuscitation, and hemodynamic support.

### Variables and definition

Demographic, clinical, and laboratory data were extracted from the medical records. This study investigated 11 complications commonly seen in HLH and sepsis, selected based on their representation of organ dysfunction in MODS according to pediatric critical care literature and common MODS-related diagnoses in our PICU practice (15, 16). These complications encompass major organ systems including hepatic (hepatic failure and hepatic injury) (2), hematologic [coagulopathy, disseminated intravascular coagulation (DIC)], cardiovascular [shock (17, 18), heart failure (19)], neurologic [central nervous system (CNS) complications], renal [acute kidney injury (AKI)] (20), respiratory [respiratory failure and acute respiratory distress syndrome (ARDS)] (21), and gastrointestinal [gastrointestinal (GI) hemorrhage]. The worst value for each complication during the entire hospital stay was used for analysis. The outcome variable was adverse outcome, defined as in-hospital death or discharge following withdrawal of advanced life-sustaining treatment. In our study population, some critically ill pediatric patients were discharged for end-of-life care at home after treatment withdrawal. This definition was used to capture the most severe adverse clinical outcomes occurring during hospitalization. Laboratory test results obtained at hospital admission and the worst value during hospitalization were extracted, including complete blood cell count, hepatic and kidney function tests, coagulation function tests, myocardial enzymes, CRP, and procalcitonin (PCT).

### Statistical analysis

Categorical variables were presented by absolute values and percentages. Continuous variables were presented by median (quartile 1 and quartile 3). Between-group comparisons for categorical variables were made using the Chi-square test or Fisher's exact test, as appropriate. Between-group comparisons for continuous variables were conducted using the Wilcoxon rank-sum test. Logistic regression was used to estimate odds ratios (OR) and 95% confidence intervals (CI) for associations between complications and outcomes, adjusting for age and sex. The distribution of the cumulative number of complications in each disease group was described using quartiles. Correlations between different complications were evaluated using the Spearman correlation coefficient. Correlation networks based on the correlation coefficients were generated using the corrr package from R. Variables with more than 30% missing data were excluded from analysis. No imputation was performed for missing data. Laboratory data were standardized using the z-score method. All hypothesis tests were two-tailed with a type 1 error rate fixed at 5%. Statistical analyses were performed using SAS 9.3 (SAS Institute, Inc., Cary, NC, USA) and R 4.1.3 (R Foundation for Statistical Computing, Vienna, Austria). Figures were generated using R and GraphPad Prism 8 (GraphPad Software, San Diego, CA, USA).

## Results

### Study population

**Figure 1:**
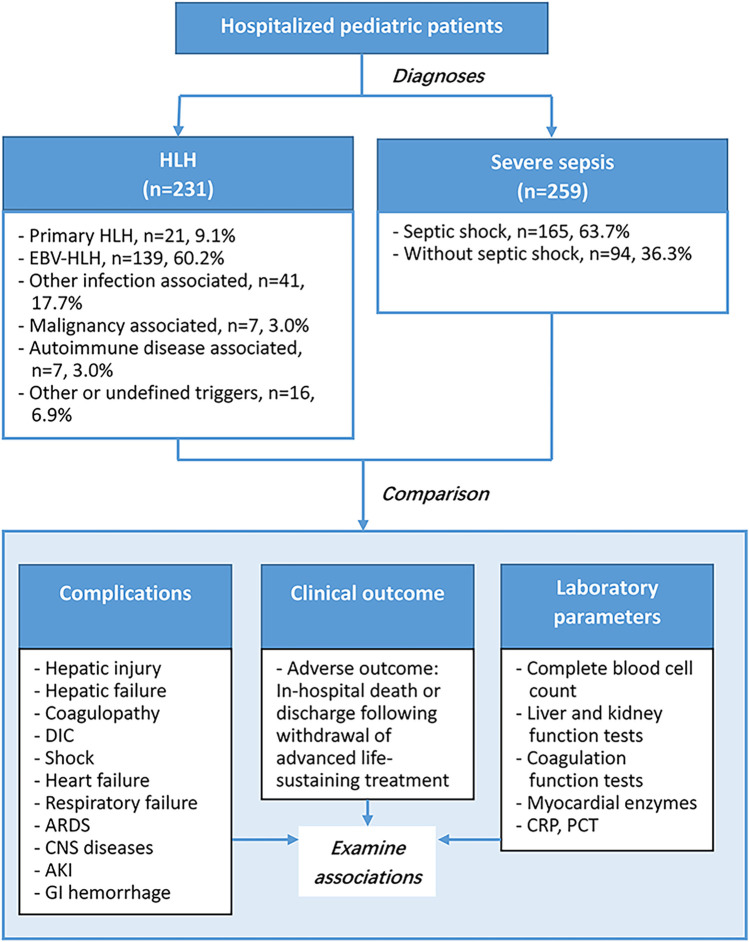
Study flow chart.

This study included 231 pediatric patients with HLH, and 259 with severe sepsis (Figure 1). The HLH group were further divided into 6 subgroups according to HLH types or potential triggers, including primary HLH (*n* = 21, 9.1%), EVB-HLH (*n* = 139, 60.2%), other infection associated HLH (*n* = 41, 17.7%), malignancy associated HLH (*n* = 7, 3%), autoimmune disease associated HLH (*n* = 7, 3%), and HLH with other or undefined triggers (*n* = 16, 6.9%). The severe sepsis group were divided into the septic shock group (*n* = 165, 63.7%) and severe sepsis without septic shock group (*n* = 94, 36.3%). The median age of patients with HLH was 3 year old, and was 1 year old among patients with sepsis (*P* < 0.0001). Among HLH group, 56.7% were male children, and in sepsis group 63.7% were male children (*P* = 0.1139). The overall adverse outcome rates in the HLH and severe sepsis groups were 15.2% and 18.9%, respectively (Table 1, *P* = 0.2693). In-hospital mortality in the HLH and severe sepsis groups was 1.73% (4/231) and 2.32% (6/259), respectively (*P* = 0.7555). For both HLH and severe sepsis group, there was no significant difference in age and sex among patients with and without adverse outcomes (Table 1).

**Table 1:** Demographic and clinical characteristics of pediatric patients with HLH and severe sepsis.

Characteristics	Total	Adverse outcome		*P*
		No	Yes	
HLH				
*N*	231	196 (84.8)	35 (15.2)	0.2693
Age, median (q1, q3)	3 (1, 5)	3 (1, 5)	2 (0, 4)	0.1147
Sex, *n* (%)				
Male	131	112 (85.5)	19 (14.5)	0.7533
Female	100	84 (84.0)	16 (16.0)	
Subtypes/potential triggers, *n* (%)				
Primary HLH	21	20 (95.2)	1 (4.8)	0.6088
EBV-HLH	139	118 (84.9)	21 (15.1)	
Other infection associated	41	34 (82.9)	7 (17.1)	
Malignancy associated	7	6 (85.7)	1 (14.3)	
Autoimmune disease associated	7	6 (85.7)	1 (14.3)	
Other or undetermined	16	12 (75.0)	4 (25.0)	
Severe sepsis				
*N*	259	210 (81.1)	49 (18.9)	0.7553
Age, median (q1, q3)	1 (0, 3)	1 (0, 3)	1 (0, 3)	
Sex, *n* (%)				
Male	165	135 (81.8)	30 (18.2)	0.6882
Female	94	75 (79.8)	19 (20.2)	
Subtypes, *n* (%)				
Septic shock	165	135 (81.8)	30 (18.2)	0.6882
Without septic shock	94	75 (79.8)	19 (20.2)	

### Incidence and adverse outcome rates of organ dysfunction

**Figure 2:**
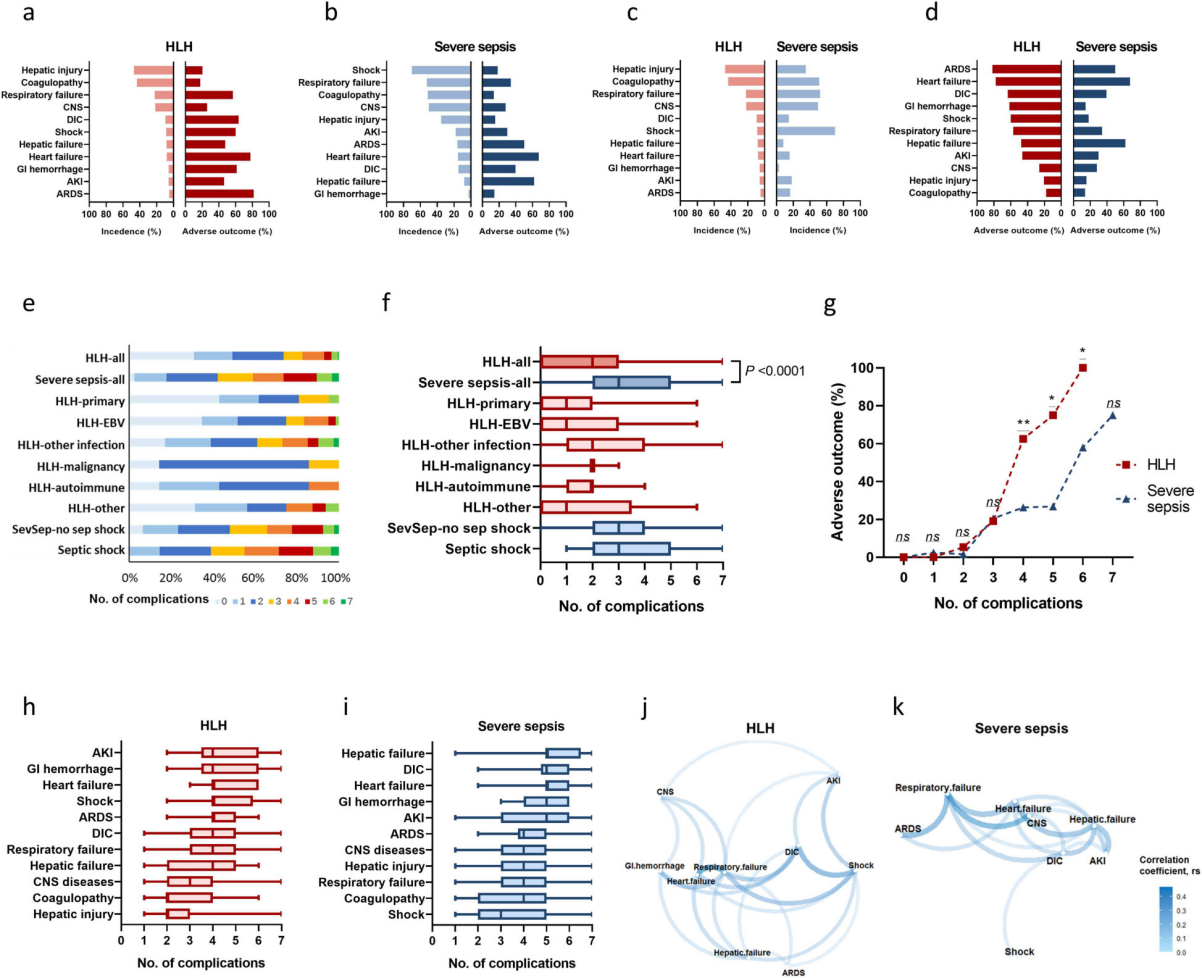
Incidences, adverse outcome rates, and clustering of complications in HLH and severe sepsis. Incidences and adverse outcome rates of complications in HLH **(a)** and severe sepsis **(b)**, sorted by incidences. Incidences of complications in HLH and severe sepsis, sorted by descending incidences in HLH (c) Adverse outcome rates of complications in HLH and severe sepsis, sorted by descending rates of adverse outcome in HLH **(d)** Distribution of the cumulated number of complications in HLH **(e)** and severe sepsis **(f)**. Adverse outcome rates according to the cumulated number of complications **(g)**. Distribution of the cumulated number of complications among those with certain types of complication in HLH **(h)** and severe sepsis **(i)**. Correlation networks of different complications among HLH **(j)** and severe sepsis **(k)**. *P < 0.05, **P < 0.01, ***P < 0.001, *ns*, non-significant. AKI, acute kidney injury; ARDS, acute respiratory distress syndrome, CNS, central nervous system; DIC, disseminated intravascular coagulation; GI, gastrointestinal; HLH, hemophagocytic lymphohistiocytosis; Sev-sepsis, severe sepsis.

Table 2 and Figures 2a–d showed the incidence and adverse outcome rates of each complication among HLH and sepsis groups. In HLH, the most prevalent complications were hepatic injury (46.8%) and coagulopathy (43.3%). In severe sepsis, the leading complications were shock (69.9%), respiratory failure (52.1%), coagulopathy (51.0%), and CNS syndromes (49.8%). Compared with the severe sepsis group, the HLH group had higher incidences of hepatic injury (46.8% vs. 35.1%, *P* = 0.0089), and had lower incidences of respiratory failure, CNS, Shock, heart failure, AKI, and ARDS (*P*-values <0.05, Table 2 and Figure 2c).

**Table 2:** Incidences and adverse outcome rates of complications in HLH and severe sepsis.

Complications	HLH (*n* = 231)		Severe sepsis (*n* = 259)		*P*
	*n* (%)	Rank	*n* (%)	Rank	
Incidence					
Hepatic injury	108 (46.8)	1	91 (35.1)	5	0.0089
Coagulopathy	100 (43.3)	2	132 (51.0)	3	0.5585
Respiratory failure	51 (22.1)	3	135 (52.1)	2	<0.0001
CNS	50 (21.6)	4	129 (49.8)	4	<0.0001
DIC	22 (9.5)	5	38 (14.7)	9	0.0827
Shock	20 (8.7)	6	181 (69.9)	1	<0.0001
Hepatic failure	19 (8.2)	7	21 (8.1)	10	0.9623
Heart failure	18 (7.8)	8	40 (15.4)	8	0.0089
AKI	13 (5.6)	9	47 (18.1)	6	<0.0001
GI hemorrhage	13 (5.6)	9	7 (2.7)	11	0.1024
ARDS	11 (4.8)	11	42 (16.2)	7	<0.0001
Adverse outcome					
ARDS	9 (81.8)	1	21 (50.0)	3	0.0885
Heart failure	14 (77.8)	2	27 (67.5)	1	0.4263
DIC	14 (63.6)	3	15 (39.5)	4	0.0711
GI hemorrhage	8 (61.5)	4	1 (14.3)	10	0.0700
Shock	12 (60.0)	5	33 (18.2)	8	0.0001
Respiratory failure	29 (56.9)	6	46 (34.1)	5	0.0047
Hepatic failure	9 (47.4)	7	13 (61.9)	2	0.3561
AKI	6 (46.2)	8	14 (29.8)	6	0.3258
CNS	13 (26.0)	9	36 (27.9)	7	0.7974
Hepatic injury	22 (20.4)	10	14 (15.4)	9	0.3627
Coagulopathy	14 (17.9)	11	13 (13.8)	11	0.4598

AKI, acute kidney injury; ARDS, acute respiratory distress syndrome; CNS, central nervous system; DIC, disseminated intravascular coagulation; GI, gastrointestinal; HLH, hemophagocytic lymphohistiocytosis.

In HLH, rates of adverse outcome were highest among patients who developed ARDS (81.8%) and heart failure (77.8%); rates of adverse outcome among patients with DIC (63.6%), GI hemorrhage (61.5%), shock (60%), and respiratory failure (56.9%) also exceeded 50%. In severe sepsis, rates of adverse outcome were highest among patients who developed heart failure (67.5%), hepatic failure (61.9%), and ARDS (50%). Rates of adverse outcome among patients with shock and respiratory failure were significantly higher in the HLH group than in the severe sepsis group (*P*-values <0.05, Table 2 and Figure 2d).

Given that hemorrhagic complications are common in HLH, we additionally analyzed pulmonary hemorrhage and intracranial hemorrhage. The incidence of pulmonary hemorrhage was 7.79% (18/231) in HLH and 5.79% (15/259) in severe sepsis (*P* = 0.483); intracranial hemorrhage occurred in 1.30% (3/231) and 2.70% (7/259), respectively (*P* = 0.437). The rates of adverse outcome for pulmonary hemorrhage were 72.22% (13/18) in HLH and 60.00% (9/15) in severe sepsis (*P* = 0.581); for intracranial hemorrhage, the rates were 0.00% (0/3) and 42.86% (3/7), respectively (*P* = 0.289). No statistically significant differences were observed between the two groups for either hemorrhage type.

### Risk of adverse outcome associated with organ dysfunction

Table 3 showed the ORs and 95% CIs of adverse outcome for each complication. In HLH, all of the investigated complications were significantly associated with adverse outcomes. In sepsis, significant associations were observed for most complications, except for shock, GI hemorrhage, hepatic injury, and coagulopathy. For the same complication, the OR of adverse outcome was higher in HLH than in severe sepsis, except for CNS syndromes. For some complications, like ARDS and DIC, the difference on OR was remarkably large between HLH and sepsis population. The OR of ARDS was 33.72 (95% CI: 6.86, 165.72, *P* < 0.0001) in patients with HLH and was 6.73 (95% CI: 3.26, 13.88, *P* < 0.0001) in patients with sepsis. The OR of DIC was 30.67 (95% CI: 9.62, 97.83, *P* < 0.0001) in patients with HLH and was 3.41 (95% CI: 1.52, 7.67, *P* = 0.0029) in patients with sepsis. When examining the cumulative effect of complications, each additional complication was associated with significantly increased risk of adverse outcome in both groups, with an OR of 4.40 (95% CI: 2.80, 6.89, *P* < 0.0001) in HLH and 2.13 (95% CI: 1.68, 2.70, *P* < 0.0001) in severe sepsis, indicating a steeper gradient of adverse outcome risk per additional complication in HLH.

**Table 3:** Associations between complications and adverse outcomes among pediatric patients with HLH and severe sepsis.

Complications	HLH		Severe sepsis	
	adOR (95%CI)^a^	*P*	adOR (95%CI)^a^	*P*
Respiratory failure	41.17 (14.96, 113.29)	<0.0001	20.92 (6.29, 69.52)	<0.0001
ARDS	33.72 (6.86, 165.72)	<0.0001	6.73 (3.26, 13.88)	<0.0001
Heart failure	33.04 (9.81, 111.33)	<0.0001	19.58 (8.71, 44.01)	<0.0001
DIC	30.67 (9.62, 97.83)	<0.0001	3.41 (1.52, 7.67)	0.0029
Hepatic failure	24.73 (6.27, 97.51)	<0.0001	9.62 (3.54, 26.15)	<0.0001
Shock	12.70 (4.65, 34.71)	<0.0001	0.85 (0.43, 1.68)	0.6361
GI hemorrhage	11.14 (3.39, 36.62)	<0.0001	0.72 (0.08, 6.18)	0.7681
Hepatic injury	6.10 (2.01, 18.48)	0.0014	1.05 (0.50, 2.19)	0.8951
AKI	5.50 (1.72, 17.54)	0.0040	2.17 (1.05, 4.49)	0.0358
Coagulopathy	3.86 (1.48, 10.05)	0.0057	0.81 (0.38, 1.72)	0.5836
CNS diseases	2.60 (1.20, 5.66)	0.0159	3.49 (1.74, 6.97)	0.0004
Each additional complication	4.40 (2.80,6.89)	<0.0001	2.13 (1.68,2.70)	<0.0001

AKI, acute kidney injury; ARDS, acute respiratory distress syndrome; CNS, central nervous system; DIC, disseminated intravascular coagulation; GI, gastrointestinal; HLH, hemophagocytic lymphohistiocytosis.

a ORs were adjusted for age and sex.

### Clustering of organ dysfunction

Figures 2e,f showed the distribution of the number of complications in each disease group. HLH had a median number of 2 (q1 = 0, q3 = 2) complications, which was significantly less than severe sepsis (median = 3, q1 = 2, q3 = 5; *P* < 0.0001). When the complications number exceeds 3, the rate of adverse outcome under the same number of complications was significantly higher in HLH than that in severe sepsis (Figure 2g). In HLH, most complications had a median number of 4 concomitant complications (Figure 2h); in severe sepsis, patients with hepatic failure, DIC, heart failure, GI hemorrhage, and AKI had a median number of 5 concomitant complications (Figure 2i). The correlation network illustrated the relationships between different complications with *P* < 0.05 (Figures 2j,k). In HLH, stronger correlations were clustered between respiratory failure, heart failure, DIC, and shock (Figure 2j). In sepsis, stronger correlations were clustered among respiratory failure, heart failure, CNS syndromes, and hepatic failure (Figure 2k).

### Laboratory characteristics

**Figure 3:**
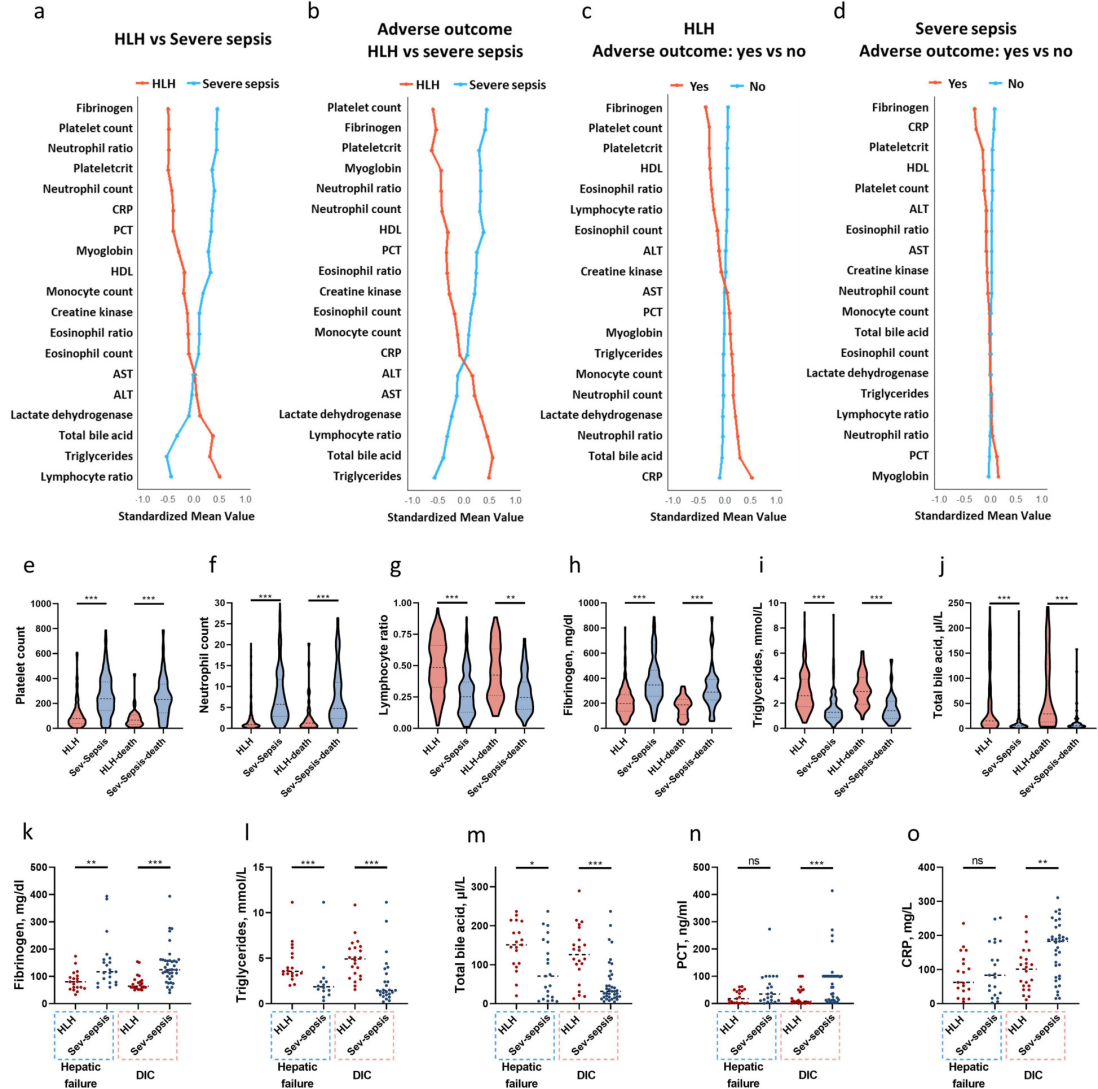
Laboratory features of pediatric HLH and severe sepsis. Standardized mean values of laboratory variables tested during hospital admission with either a >1.5 or <0.667 fold change difference (*P* < 0.001) between HLH and severe sepsis **(a)**, between patients with adverse outcomes in HLH and severe sepsis **(b)**, between patients with and without adverse outcomes in HLH **(c)**, and between patients with and without adverse outcomes in severe sepsis **(d)** The earliest tests results of platelet count **(e)**, neutrophil count **(f)**, lymphocyte ratio **(g)**, fibrinogen **(h)**, triglycerides **(i)**, and total bile acid **(j)** levels among patients with HLH and sepsis. Worst values of fibrinogen **(k)**, triglycerides **(l)**, total bile acid **(m)**, PCT **(n)**, and CRP **(o)** among those with hepatic failure and DIC. *P < 0.05, **P < 0.01, ***P < 0.001, *ns*, non-significant. ALT, alanine aminotransferase; AST, aspartate aminotransferase; CRP, C-reactive protein; HLH, hemophagocytic lymphohistiocytosis; PCT, procalcitonin; Sev-sepsis, severe sepsis.

Figure 3a showed the standardized means of laboratory variables tested during hospital admission with either a >1.5 or <0.667 fold change difference between HLH and severe sepsis (*P* < 0.001). Figures 3b–d presents these variables according to outcome and disease groups. Compared to severe sepsis, HLH is characterized by lower fibrinogen, platelet count, neutrophil count, and higher lymphocyte ratio, triglycerides, and total bile acids (Figures 3a,e–j). In both HLH and severe sepsis conditions, the most pronounced differences between patients with and without adverse outcomes were noted in the levels of fibrinogen (Figures 3c,h). However, a more substantial decline in fibrinogen levels was observed in HLH patients with adverse outcomes compared to those with severe sepsis (Figures 3b,h). We also compared the worst laboratory values in patients with HLH and sepsis, specifically among those with hepatic failure or DIC. Patients developed hepatic failure or DIC were characterized by decreased fibrinogen and elevated triglycerides and total bile acids (Figures 3k–m), while patients with severe sepsis and developed DIC were characterized by elevated PCT, and CRP (Figures 3n,o). The worst value of ALT, AST, and D-dimer showed no significant differences between HLH and severe sepsis among those with hepatic failure or DIC (*P* > 0.05).

## Discussion

Our data showed both HLH and severe sepsis exhibited high incidences of severe complications, which were associated with an increased risk of adverse outcomes. This study applied the 2005 International Pediatric Sepsis Consensus Conference definitions (2), which remained standard practice during our study period (2018–2023). We acknowledge that sepsis definitions have evolved, with the 2016 Sepsis-3 consensus (22) eliminating the term “severe sepsis” in adult populations and the 2024 Phoenix criteria (4) introducing updated pediatric-specific definitions. While this may affect direct comparability with studies using newer definitions, we believe our findings regarding organ dysfunction patterns remain clinically relevant. The Phoenix criteria emphasize organ dysfunction assessment through the Phoenix Sepsis Score, which aligns conceptually with our focus on specific organ dysfunction patterns. Future studies validating our findings using the Phoenix criteria would strengthen the generalizability of our conclusions to current clinical practice, though we expect the fundamental differences in organ dysfunction patterns between HLH and severe sepsis to persist across different diagnostic frameworks.

Our study focused on organ dysfunction patterns that constitute MODS, comparing how these manifest differently in HLH vs. severe sepsis. Key findings that distinguish these two conditions include: First, incidence patterns showed that HLH had lower incidences of most organ dysfunctions compared to severe sepsis, but with similar or higher adverse outcome rates. Second, HLH patients developed fewer complications overall (median 2 vs. 3), but exhibited higher adverse outcome rates per complication. Third, each additional complication increased risk of adverse outcome more steeply in HLH (OR = 4.40, 95% CI: 2.80, 6.89) than in severe sepsis (OR = 2.13, 95% CI: 1.68, 2.70). Fourth, complication networks differed substantially, with HLH featuring DIC and shock as hub nodes, while severe sepsis was characterized by CNS complications and hepatic failure as central nodes. Fifth, laboratory profiles distinguished the two conditions, with HLH showing lower fibrinogen, platelet count, and neutrophil count, along with higher lymphocyte ratio and triglycerides, whereas severe sepsis exhibited elevated CRP and PCT. For most organ dysfunctions that we investigated, HLH showed lower incidences than that of severe sepsis, but with similar or higher risk of adverse outcome. Under the same number of complications, HLH had a higher adverse outcome rates than severe sepsis; patients with severe sepsis tended to develop more complications, resulting in similar overall adverse outcome rates for these two conditions. These findings suggest different strategies for improving prognosis in these two conditions. For HLH, preventing progression to severe organ dysfunction through early and aggressive disease-specific therapy appears critical, whereas for severe sepsis, limiting the accumulation of multiple organ failures through timely infection control and organ support may be more important.

HLH and sepsis both involve systemic inflammation leading to organ dysfunction, yet they differ significantly in their underlying mechanisms (23, 24). HLH is characterized by immune dysregulation, primarily manifesting as an uncontrolled and excessive immune response, often triggered by infections, autoimmune disorders, or malignancies (1, 24). This is marked by the over activation of T-lymphocytes and macrophages, leading to a “cytokine storm” and subsequent further organ dysfunctions. In contrast, sepsis is characterized by an initial pro-inflammatory activation with a dysregulated anti-inflammatory response, which leads to endothelial dysfunction, coagulation abnormalities, and a generalized, excessive inflammatory response known as the SIRS (25, 26). As the specific pathways that lead to organ dysfunction in HLH and sepsis can vary, their manifestations also differ. The associations between different complications may reflect their similarity in vulnerability to certain types of attacks or damage. Consistent with findings from other studies, our data revealed that at hospital admission, patients with HLH were characterized by abnormal hepatic and blood cell tests, while those with severe sepsis exhibited elevated levels of CRP and PCT (11). As the diseases progressed, we observed differences in the incidence of complications between HLH and severe sepsis, as well as in the correlation networks among these complications. Although respiratory failure and heart failure are key nodes in both conditions, the complication network in HLH was characterized by hub nodes of DIC and shock, while in severe sepsis, CNS complications and hepatic failure were identified as hub nodes. These findings suggest research directions exploring why certain complications are more central in one condition than in another. Such exploration could yield insights into the molecular and cellular mechanisms driving these diseases. Moreover, longitudinal research is needed to trace the development of MODS, identifying early signs of key complications. Aggressive interventions informed by these signs could significantly contribute to disease management strategies.

Among patients who developed shock or respiratory failure, rates of adverse outcome in HLH (60.0% and 56.9%, respectively) were significantly higher compared to severe sepsis (18.2% and 34.1%). For other complications, except hepatic failure and CNS complications, rates of adverse outcome were higher in HLH, though not statistically significant. Since the patients were from the same hospital with identical medical care standards, and HLH cases had fewer complications than sepsis, the reason for the higher adverse outcome rates observed in HLH may reflect greater difficulty in managing complications when they develop in the context of the underlying pathophysiology of HLH, though the precise mechanisms remain to be elucidated. The treatment of HLH and severe sepsis both require source control and supportive care. The HLH-specific therapy follows the HLH-94 treatment protocol, which includes dexamethasone, etoposide, intravenous immunogloblin, cyclosporine A, and intrathecal methotrexate, varying based on severity, symptoms, and treatment response. Severity stratification, based on organ dysfunction, has been applied in HLH and is used to guide treatment (27). Salvage therapies like ruxolitinib and epavizumab have also been proven effective (27). Although HLH-specific therapy has improved the outcomes of HLH, adverse outcome rates remain high. Patients may die before completing or even starting the treatment, due to non-response to treatment, deterioration after initial response, or disease relapse. Irreversible organ dysfunction and failure in HLH may result from uncontrolled systemic immune dysregulation or from the immunosuppressive treatment itself. Chemotherapy has the potential to induce organ injury. Furthermore, while immunosuppressive treatments that inhibit uncontrolled inflammation are desirable, immune reconstitution is also necessary to resolve infections (28). Balancing these aspects is crucial for effective immune reconciliation. We also noticed that in HLH, patients with hepatic failure and DIC were characterized by decreased fibrinogen, along with elevated triglycerides and total bile acids. In severe sepsis, patients with DIC exhibited elevated levels of PCT and CRP. However, ALT, AST, and D-dimer showed no significant differences between the two diseases. For the same organ, the characteristics of dysfunction differed between the two diseases, suggesting that targeted therapy to prevent or treat organ dysfunction should vary by disease. Given the high adverse outcome rates associated with organ dysfunction in both HLH and sepsis, developing novel therapies grounded in a deeper understanding of each disease's mechanism is necessary.

To date, this is the first study to compare patterns of organ dysfunction in HLH and severe sepsis. Patients with either of these two diseases were drawn from the same population, enhancing the comparability of the two groups and minimizing the impact of selection bias. Our analysis not only compared the incidence and adverse outcome rates but also investigated the accumulated number of complications and the correlation networks among these complications. This approach has yielded insights that could inform clinical management strategies and guide future research in pediatric HLH and sepsis. This study has several limitations. First, as a single-center study with a limited sample size, the representativeness of the study population and the generalizability of the findings require further validation. Second, we acknowledge using the 2005 pediatric sepsis definitions rather than more recent criteria; future studies should validate our findings using updated definitions such as the Phoenix criteria to ensure applicability in contemporary clinical settings. Third, our primary outcome combined in-hospital mortality and withdrawal of life-sustaining treatment as a composite outcome measure of adverse events, which may be influenced by subjective decisions regarding treatment withdrawal and may limit direct comparability with studies that use standard mortality as the outcome measure. However, we reviewed medical records and confirmed that all cases of treatment withdrawal occurred at the terminal stage, reflecting decisions to forgo therapy when prognosis was extremely poor. This is consistent with previous research showing that discharge to palliative care resulted in fewer deaths in hospital, with a greater proportion of deaths occurring at home or in a hospice (29). Therefore, we consider this composite outcome to represent the worst-case clinical scenario. Fourth, although investigating the sequence of complications is crucial for identifying targets for early intervention, this aspect was not examined in this study. The development network of different complications is dynamic, complex, and characterized by reciprocal causation; therefore, this research demands sufficient data to monitor the progression of various complications and sophisticated statistical models. Fifth, we were unable to compare cytokine profiles between the two groups due to substantial missing data and the use of different cytokine detection kits across the study population, which limited comparability. Future prospective studies with standardized cytokine measurements could provide valuable insights into the immunological mechanisms underlying the observed differences in organ dysfunction patterns and adverse outcome rates. Finally, while this study observed differences in organ dysfunction patterns between HLH and severe sepsis, it did not explore the underlying reasons. Mechanistic studies are needed to elucidate these differences and to explore new therapeutic approaches.

## Conclusion

Both HLH and severe sepsis exhibited high incidences of organ dysfunction leading to MODS. The incidence, adverse outcomes, and clustering patterns of organ dysfunction differed between HLH and severe sepsis. Patients with HLH generally had a lower incidence of organ dysfunction and fewer complications than those with severe sepsis. However, HLH patients who developed organ dysfunction had higher adverse outcome rates compared to their counterparts with severe sepsis. In severe sepsis, a large proportion of patients developed multiple organ dysfunctions, which was also associated with high adverse outcome rates. These findings suggest different management priorities: in HLH, preventing individual organ dysfunction is crucial given the strong association between organ dysfunction and adverse outcomes; in severe sepsis, preventing the cascade of multiple organ failures is essential. These insights could inform clinical management strategies and guide future research in pediatric HLH and sepsis, irrespective of evolving diagnostic criteria for these conditions.

## Data Availability

The original contributions presented in the study are included in the article/Supplementary Material, further inquiries can be directed to the corresponding authors.
